# Optimising gynaecological surgical care for elite female athletes: a narrative review

**DOI:** 10.3389/fspor.2026.1746638

**Published:** 2026-07-08

**Authors:** George Lockett, Konstantinos S. Kechagias, Amelia Thomson, Zain Velji, Nina Cooper, Jennifer Barcroft, Benjamin P. Jones, Jonny R. Stephens, Sanooj Soni, Michael Dooley, Guy Evans, Noel Pollock, Kirsty Munro, Maya Al-Memar, Srdjan Saso

**Affiliations:** 1 Hammersmith Hospital, Imperial College NHS Trust, London, United Kingdom; 2 Department of Metabolism, Digestion and Reproduction, Imperial College London, London, United Kingdom; 3 Lister Fertility Clinic, The Lister Hospital, London, United Kingdom; 4 Department of Critical Care, Imperial College Healthcare NHS Trust, London, United Kingdom; 5 Division of Anaesthetics, Pain Medicine and Intensive Care, Imperial College, London, United Kingdom; 6 King Edward VII’s Hospital, London, United Kingdom; 7 Lawn Tennis Association, Roehampton, London, United Kingdom; 8 Institute of Sport, Exercise and Health, University College London, London, United Kingdom; 9 Royal Infirmary of Edinburgh, Edinburgh, United Kingdom

**Keywords:** elite female athletes, gynaecological surgery, multidisciplinary rehabilitation, peri-operative care, return to sport

## Abstract

**Background:**

With the rapid growth of women's elite sport, there is an increasing need to optimise healthcare for female athletes. These athletes can present unique clinical challenges, including high physical demands, altered energy availability, increased rates of pelvic floor dysfunction, and sport-related psychological pressures. Gynaecological surgery may significantly disrupt training and competition schedules, impacting both short- and long-term performance.

**Objectives:**

This narrative review provides a practical, evidence-based framework to support clinicians in delivering tailored peri-operative care for female athletes undergoing gynaecological surgery.

**Key findings:**

Drawing from current literature and multidisciplinary expertise, the review outlines key considerations across the pre-, intra-, and post-operative phases. It emphasises the importance of pre-operative assessment of menstrual health, bone density, nutritional status, and psychological readiness, particularly in athletes at risk of Relative Energy Deficiency in Sport (RED-S). Intra-operatively, surgical techniques should account for anatomical variations in lean athletes, and measures should be taken to minimise complications such as neuropathy, wound breakdown and delayed recovery. Post-operative rehabilitation requires a coordinated, multidisciplinary approach integrating physiotherapy, nutrition, pain management, and psychological support to facilitate a safe and timely return to sport.

**Conclusions:**

This review highlights the need for athlete-specific surgical strategies that align with the physiological and performance demands of elite sport. Future studies are essential to inform sport-specific guidelines and optimise outcomes for this understudied population.

## 1 Introduction

Elite women's sport is experiencing a period of rapid growth with the number of female athletes steadily increasing over the last two decades (1). This increase is also evident in the expanding media coverage and the growing number of spectators attending women's sporting events.^1^ With the growing popularity of women's sport, there will be a corresponding increase in elite athletes requiring tailored and specialised gynaecological surgical care.

Despite these trends, the field of Sports Gynaecology is still in its infancy. Whilst there is a greater understanding and appreciation of the unique physiological changes and gynaecological presentations that can affect elite athletes (2), there is a paucity of guidance or literature on managing the athlete requiring gynaecological surgery (1). Alongside the physical demands of their sport, female athletes face distinct challenges related to gynaecological health that can significantly impact their well-being and performance. Gynaecological conditions such as pelvic floor disorders, endometriosis, ovarian cysts, and menstrual irregularities are prevalent among female athletes, posing both physiological and psychological hurdles to their athletic careers (3).

This article aims to provide a comprehensive review of pre-, intra- and post-operative care of the elite female athlete. Moreover, we propose a clinical framework outlining how different gynaecological surgical approaches, namely hysteroscopy, laparoscopy and open surgery, can be tailored to optimise the management and surgical outcomes of this unique population emphasising the importance of multidisciplinary care.

The study drew upon peer-reviewed literature, consensus statements, clinical guidelines, and expert opinion relevant to the peri-operative management of elite female athletes undergoing gynaecological surgery. Literature searches for relevant articles were conducted in PubMed, Embase, and Scopus using combinations of keywords including “elite athlete”, “female athlete”, “gynaecology”, “gynaecological surgery”, “peri-operative care”, “return to play”, and “sports medicine”. Additional relevant studies were identified through manual screening of reference lists from key articles and guidelines. A summary of the search strategy, including databases, search terms, and search dates, is provided in the Supplementary Table S1.

Studies were selected based on their relevance to the peri-operative care of female athletes and/or the optimisation of surgical outcomes, rehabilitation, and return to sport. Given the limited availability of athlete-specific evidence, the review also incorporated relevant literature from broader surgical, anaesthetic, rehabilitation, and sports medicine populations where findings were considered applicable to elite female athletes. The evidence was synthesised narratively rather than through formal systematic review methodology, and recommendations were formulated using a pragmatic assessment of the available literature.

## 2 Clinical and psychological considerations in the surgical care of the elite female athlete

Managing elite female athletes involves distinct challenges beyond routine gynaecological care. Intense training, nutritional constraints, and performance pressures can exacerbate underlying conditions, necessitating a nuanced understanding of their physiological, anatomical, and psychological profiles to guide effective surgical and peri-operative care.

### 2.1 Relative energy deficiency in sport (RED-S)

Relative Energy Deficiency in Sport (RED-S) is a multifactorial syndrome arising from an imbalance between dietary energy intake and energy expenditure, wherein insufficient energy remains for optimal physiological function (4). RED-S affects a significant proportion of high-performance athletes and can result in health consequences including menstrual dysfunction, subfertility and changes in bone mineral density. In particular, the prevalence of menstrual disorders in athletes with lower BMIs is higher than that of athletes with a BMI in the normal range (5).

### 2.2 Pelvic floor dysfunction in athletes

Pelvic floor dysfunction (PFD) is prevalent among elite female athletes, particularly those involved in high-impact or load-bearing sports (6). A 2018 systematic review and meta-analysis reported the prevalence of urinary incontinence at 36% in this population with peak prevalence reaching 76% (7).

The pathophysiology of PFD in athletes is multifactorial. Contributing factors include repeated elevations in intra-abdominal pressure, and over-recruitment or fatigue of the pelvic floor. Prolonged exposure to these stressors may predispose athletes to microtrauma, neuromuscular dysfunction, or compensatory patterns that eventually manifest as symptoms (8). Pre-existing PFD should therefore be actively identified before surgery, as it may influence both early post-operative recovery and the sequencing of rehabilitation. Athletes with symptoms such as urinary incontinence, pelvic heaviness, pain, or impaired load tolerance may require early pelvic health physiotherapy input, with rehabilitation prioritising breath control, pressure management, graded pelvic floor loading, and sport-specific return-to-impact progression (9).

### 2.3 Psychological considerations and performance anxiety

Elite sport also entails significant psychological burden and sport-related anxiety is another important consideration (10). According to Mann et al., the three most frequently discussed injury-related topics between physician and athlete include fear of reinjury, anxiety surrounding surgery, and frustration with the pace of recovery or rehabilitation (11). These findings highlight the importance of adopting a holistic and athlete-centered approach, in which psychological well-being is addressed alongside physical health. Dergaa et al., in a recent paper, supports a multidisciplinary sports medicine model in which psychological support, athlete education, communication, monitoring and individualised recovery planning are treated as core components of safeguarding elite athlete health and performance, rather than optional adjuncts (12).

A further consideration is athletic identity disruption. For elite athletes, enforced absence from training and competition may represent not only a physical limitation but also a temporary loss of role, structure, autonomy, and belonging. This is particularly relevant in the peri-operative period, when athletes may be separated from their usual coaching environment, peer group, performance routines, and competitive goals. Athletic identity disruption may therefore compound anxiety, frustration, low mood, or engagement with rehabilitation (13). A strong association has been also described between disordered eating patterns and heightened anxiety levels in female athletes, indicating a bidirectional relationship between psychological and physiological stressors (14). The peri-operative period may amplify these vulnerabilities, particularly as athletes may be separated from their familiar routines, coaching structures, and support systems during recovery. To mitigate these risks, early input from sports psychologists should be considered a standard component of peri-operative care.

### 2.4 Endometriosis and surgical care

Endometriosis warrants particular consideration within the context of gynaecological surgery in elite female athletes, as it represents one of the most common indications for operative intervention in reproductive-aged women (15). Despite its prevalence, there is a paucity of evidence specifically evaluating the peri-operative management, recovery trajectories, and return-to-play (RTP) outcomes of elite athletes undergoing surgery for endometriosis. Consequently, many aspects of clinical decision-making are extrapolated from the broader gynaecological and surgical literature. Surgical management, most commonly through laparoscopic excision or ablation of endometriotic lesions, may present unique challenges owing to the variable extent of disease, potential involvement of pelvic organs, and the complexity of some procedures (16). Careful pre-operative planning is essential to balance symptom relief and disease control against the anticipated recovery period and potential disruption to training and competition schedules. Athletes undergoing surgery for endometriosis may require individualised rehabilitation plans, particularly following extensive pelvic dissection, bowel or urinary tract involvement, or repeat procedures (Supplementary Table S2). Furthermore, the chronic and recurrent nature of the disease means that surgery should be considered as part of a broader multidisciplinary management strategy that may include hormonal therapies, pain management, nutritional support, physiotherapy, and psychological support. Given the high prevalence of endometriosis and its frequent role as an indication for surgery, clinicians caring for elite female athletes should be familiar with the peri-operative considerations and recovery challenges associated with its management.

## 3 Surgery- to operate or not to operate

Before embarking on surgical intervention, the gynaecologist must consider whether operative management is warranted. This process begins with a thorough history and physical examination, supported by appropriate investigations, including blood tests and diagnostic imaging. Crucially, the consultation should provide a platform for shared decision-making, allowing the athlete to express her priorities, expectations, and concerns. With athlete consent, there may be an important role for the athlete's coach/manager in the shared decision-making process to further consider contextual factors such as training and competition schedules (17). The clinician should however be mindful of the potential for conflicting interests from performance stakeholders (18). Medical and conservative therapies should ordinarily be exhausted prior to committing to surgery. These will include pharmacological treatments, physical therapy, and watchful waiting, where clinically appropriate.

Ultrasound imaging plays a central role as a diagnostic tool in the field of gynaecology and its use is fundamental in the evaluation of common gynaecological symptoms such as pelvic pain and heavy menstrual bleeding (19). For the ultrasound evaluation of adnexal lesions, Assessment of Different NEoplasias in the adneXa (ADNEX) model and the International Ovarian Tumour Analysis (IOTA) two-step strategy are validated tools to help differentiate between benign and malignant lesions (20). Endometrial pathology can be assessed and described on ultrasound by using International Endometrial Tumor Analysis (IETA) terminology and the IETA-1 model can help to differentiate between benign and malignant endometrial pathologies in women both with and without abnormal uterine bleeding (21). Myometrial conditions can be evaluated and described based on ultrasound with Morphological Uterus Sonographic Assessment (MUSA) terminology (22). The different phenotypes of endometriosis can be described and evaluated on ultrasound utilising the International Deep Endometriosis Analysis (IDEA) group's approach (23). These models offer an evidence-based and systematic approach to describing imaging findings which can be interpreted by the operating gynaecologist to plan and assess the need and urgency for surgery.

Overall, while established clinical and imaging frameworks remain central to surgical decision-making, the management of elite athletes requires additional consideration of sport-specific factors. The timing of surgery should be individualised and balanced against competition schedules, training periodisation, symptom burden, risk of disease progression, and the anticipated impact of postoperative recovery on athletic participation and performance. Consequently, decisions regarding operative vs. conservative management should be made within a multidisciplinary framework that incorporates both clinical and athletic objectives. Once the decision for surgery has been made, the focus should shift to pre-operative care. 

## 4 Pre-operative management

**Figure 1:**
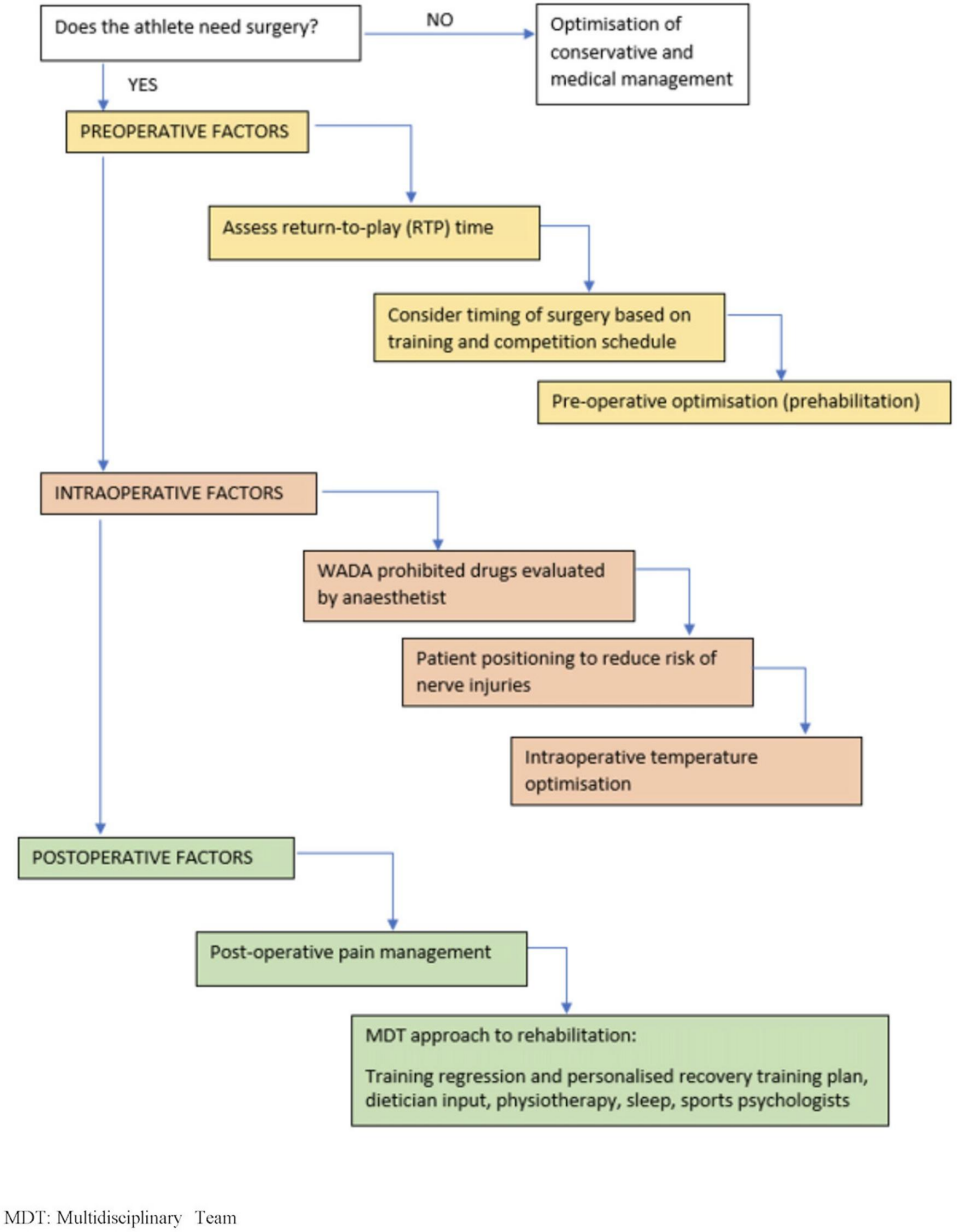
Surgical optimization flowchart.

Pre-operative optimisation of any patient prior to surgery is a cornerstone of the principles of enhanced recovery.^2^ These principles can be modified to fit the needs of elite female athletes (Figure 1).

### 4.1 Timing of surgery

The timing of surgery is a critical factor. RTP time encompasses the time between surgery and a full return to competition-level fitness. Minor gynaecological surgery such as hysteroscopic procedures and simple laparoscopic procedures will carry a short RTP time and thus have a less detrimental impact on training and competition (Supplementary Table S2). As a result, these procedures allow for greater flexibility in surgical scheduling. Conversely, major surgery which carries a longer RTP time of weeks to months will impact an athlete's ability to train and compete. Consequently, such operations demand more strategic planning and coordination with the athlete's performance goals, seasonal commitments and, potentially, contractual status. Where possible, gynaecologists should consider scheduling elective procedures with prolonged RTP times during the off-season to minimise disruption. In the interim, conservative or medical management of gynaecological conditions may be appropriate until an optimal surgical window becomes available.

The menstrual cycle may represent an additional consideration when planning elective surgery in elite female athletes. Fluctuations in oestrogen and progesterone concentrations throughout the cycle have been associated with changes in pain perception, ligamentous laxity, neuromuscular control, inflammatory responses, and haemostatic function (24). Some studies in sports medicine have suggested that these physiological variations may influence injury risk, performance, and recovery, although findings remain inconsistent and the overall quality of evidence is variable (25). The follicular phase has been hypothesised to represent a potentially favourable period for surgery because of lower progesterone concentrations, more stable hormonal profiles, and theoretical advantages relating to tissue healing, pain sensitivity, and postoperative rehabilitation. Conversely, the late luteal phase may be associated with increased fluid retention, altered pain perception, and premenstrual symptoms that could negatively affect peri-operative wellbeing and early recovery. However, direct evidence linking menstrual cycle phase to gynaecological surgical outcomes, complication rates, or RTP timelines is currently lacking. Therefore, while menstrual cycle timing may be considered as part of individualised pre-operative planning when clinically feasible, it should not delay necessary treatment or take precedence over other important factors, including disease severity, surgical urgency, training schedules, and athlete preference.

### 4.2 Pre-operative optimisation—prehabilitation

RTP will be adversely affected if there are any peri-operative surgical complications. Prehabilitation refers to the interventions taken prior to major surgery that can improve post-operative outcomes for the patient and spreads the emphasis of rehabilitation from a solely post-operative construct to one that begins before surgery (26). In elite athletes, however, the objectives of prehabilitation may differ from those of the general surgical population. Rather than focusing primarily on increasing baseline functional capacity, prehabilitation should aim to preserve existing physiological performance, minimise deconditioning during the peri-operative period, and optimise sport-specific factors that may influence recovery, including iron deficiency, pelvic floor dysfunction, menstrual health, and RED-S. Particular attention should be given to athletes with low energy availability, as restoration of adequate nutritional status and energy balance may be required before elective surgery to support wound healing, recovery, and RTP.

Exercise prior to surgery reduces post-operative complication rate and promotes earlier restoration of functional status (27). Nutritional, psychological, and behavioural interventions are considered part of prehabilitation and addressing these has been shown to improve functional recovery (28). Input for each patient will be unique, but a routine starting point would be optimisation of any haematological or biochemical markers which can be identified through a set of routine blood tests (29). Frequent or chronic NSAID use should be specifically assessed, as elite athletes may use these agents regularly to manage training-related pain or injury. Depending on the agent, dose, timing, and patient-specific risk factors, NSAIDs may have implications for platelet function, renal perfusion under general anaesthesia, gastrointestinal risk, and peri-operative bleeding (30). Where relevant, NSAID cessation, substitution, or continuation should be individualised in discussion with the anaesthetic and surgical teams, while ensuring that alternative analgesic strategies are available to avoid poorly controlled pain during the peri-operative period.

Of note, female athletes tend to experience a greater incidence of iron deficiency with low energy intake, vegetarian diets and endurance exercise proposed as contributing factors (31). There is a clear link between iron regulation and exercise and specific criteria have been previously described to classify the various stages of iron deficiency in athletes (32). Standardisation of blood collection procedures for pre-operative assessment is also essential. Factors such as the time of day, hydration status, and recent physical activity should be carefully considered, as they can significantly influence haematological parameters (33). Notably, muscle-damaging exercise, particularly eccentric activity, should be avoided ideally for 2 days prior to testing, as it can provoke elevated levels of systemic inflammation, potentially altering the blood profile obtained (34).

### 4.3 Nutrition

Nutritional status is a critical determinant of surgical outcomes. The dietary practices of elite athletes differs from the general population (35). Optimising nutrition in the pre-operative period can significantly influence wound healing, immune response, muscle preservation, and overall recovery, and should therefore be considered an essential component of peri-operative planning (36). In athletes at high risk of RED-S, referral to a sports nutritionist is recommended to restore adequate energy balance prior to elective surgery (37). In the peri-operative and post-operative period, the athlete may be at risk of RED-S due to a self-imposed significant reduction in energy intake as they are not undertaking their usual training. A peri-operative discussion on the importance of adequate energy intake for recovery can mitigate this risk.

Macronutrient adequacy is another fundamental aspect of pre-operative preparation. Protein intake must be sufficient to support tissue repair and preserve lean body mass during periods of reduced physical activity. Recommendations suggest that athletes consume between 1.4 and 2.0 grams of protein per kilogram of body weight per day, with an emphasis on high-quality, leucine-rich sources to stimulate muscle protein synthesis (38). Carbohydrate intake should support immune competence and metabolic resilience; while training demands may be reduced pre-operatively, carbohydrate restriction is not advised, as it may exacerbate the physiological stress of surgery (39). Dietary fats, particularly those rich in omega-3 fatty acids, contribute to cell membrane integrity and inflammation modulation, and should be included in appropriate quantities to support recovery (40).

In addition, attention must also be paid to micronutrient sufficiency. Vitamin D, calcium and magnesium are essential for bone health and neuromuscular function, and low levels may impair healing and increase the risk of complications (41). Assessment of vitamin D status should be performed in athletes with limited sun exposure or known risk factors for deficiency. Female athletes are at greater risk of vitamin D deficiency relative to males, potentially related to UV blocking skin moisturiser or make up (42). Magnesium levels have been shown to be commonly deficient amongst athletes (43). Other nutrients such as vitamin C, vitamin A, and zinc are known to support immune function and tissue regeneration, and while supplementation is not routinely required in well-nourished athletes, these should be considered in those with restricted or suboptimal diets (44).

Emerging evidence also highlights the potential role of immunonutrition; specialised nutritional formulations containing compounds such as arginine, omega-3 fatty acids, and nucleotides, in enhancing immune competence and reducing post-operative morbidity (45). While the data are more robust in gastrointestinal and oncological surgical populations, immunonutrition may hold promise for athletes undergoing major gynaecological procedures, though further research is needed to substantiate its use in this specific cohort (46).

### 4.4 Venous thromboembolism risk

Venous thromboembolism (VTE) prevention warrants particular consideration in elite female athletes undergoing gynaecological surgery, especially among those using hormonal contraception. Combined hormonal contraceptives are commonly utilised by athletes for contraception, menstrual suppression, cycle manipulation around training and competition schedules, and the management of menstrual-related symptoms. However, oestrogen-containing contraceptives are associated with an increased risk of VTE through their prothrombotic effects on coagulation pathways. In the peri-operative setting, this risk may be compounded by additional factors including surgical tissue injury, inflammation, reduced mobility, dehydration, and long-distance travel, all of which are frequently encountered within elite sport. Previous literature has highlighted hormonal contraceptive use as an important contributor to hypercoagulability in female athletes and recommends careful assessment of individual thrombotic risk factors when planning treatment (47). In athletes with additional VTE risk factors, including a personal or family history of thrombosis, thrombophilia, prolonged immobilisation, major surgery, or previous VTE, consideration should be given to peri-operative risk stratification and implementation of appropriate thromboprophylaxis according to established surgical guidelines (47, 48). Decisions regarding continuation or temporary discontinuation of combined hormonal contraception should be individualised, balancing thrombotic risk against contraceptive needs, menstrual management requirements, and the potential impact on training and competition schedules. Close collaboration between gynaecologists, anaesthetists, haematologists, sports physicians, and the athlete is recommended to optimise peri-operative safety and minimise disruption to athletic performance.

## 5 Intra-operative management

The gynaecologist should maintain a comprehensive perspective on the intra-operative management of elite athletes to ensure that all members of the surgical team are informed of modifiable aspects of care that may enhance postoperative recovery and optimise clinical outcomes. This involves discussion and liaison with the anaesthetist and the wider theatre team. The Preoperative Briefing (Team Brief) is a common time to discuss intra-operative management and any anticipated challenges, and it can be used as forum to empower all members of the surgical team managing the female athlete (Figure 1) (49).

### 5.1 Anaesthetic considerations

Anaesthetists will require a specific approach to optimising intraoperative and postoperative anaesthesia in this subset of patients. It is necessary to have the right choice of anaesthetic technique, pain management options, understanding of specific physiologic adaptations of the athlete, and knowledge of prohibited substances (50). Extreme training induces cardiovascular, respiratory and cerebral autoregulation changes, as well as extremes of body composition and muscle mass. The most common benign ECG findings in athletes are bradycardia, isolated left ventricular hypertrophy on voltage criteria and early repolarisation and the anaesthetist should be able to identify that these are normal features in the athlete's heart (50). Whilst these are a normal physiological adaptations to rigorous training regimes, they must be acknowledged and understood to avoid unnecessary concern and or overtreatment.

The anaesthetist taking care of an athlete should be prudent about administering drugs restricted by the World Anti-Doping Agency (WADA), outlined on their “Prohibited List”. This comprises performance enhancing substances and methods that are prohibited either at all times, in competition or in particular sports only. Anaesthetists treating elite athletes should be cognisant that this list is reviewed and updated annually. For example, in 2024, Tramadol was added (51). When the clinical situation requires any medication on the prohibited list of WADA, the athlete and their team should be informed, ideally in writing. Athletes can apply for “Therapeutic Use Exemptions” (TUEs) for authorisation to use prohibited substances; in emergency treatment this can be applied for in retrospect (52). Some substances are only prohibited in-competition, defined as ‘The period commencing at 23:59 on the day before a Competition in which the athlete is scheduled to participate through the end of such Competition and the Sample collection process related to such Competition (53).

### 5.2 Patient positioning

Careful consideration of patient positioning is important in the elite athlete. Lithotomy position is commonly used in gynaecological operating and is associated with a 1.5% risk of developing lower limb neuropathy including obturator, lateral femoral cutaneous, sciatic and peroneal nerve neuropathies (54). Prolonged positioning in lithotomy is the main risk factor for these neuropathies, and although most resolve soon after surgery, this is an undesirable outcome for the elite athlete.

Minimising time in lithotomy and the use of appropriate padding can help to reduce their incidence (55). Risk of neuropathies of the lower extremity can be reduced by ensuring cushioning at the fibular neck, avoiding hip flexion >90 degrees in lithotomy, and avoiding extremes of abduction and external rotation of the hip joint (56). Neuropathy of the upper limb can be associated with laparoscopic or robotic-assisted procedures performed in the Trendelenburg and Lloyd-Davies position (57). Rises in intraocular pressure caused by Trendelenburg's position will also increase the risk of perioperative visual loss (58). These risks can be mitigated for by avoiding the use of shoulder supports and reducing intraoperative sliding using non-slip pads. Thus, the extent of tilt should be kept to a minimum and the duration of any procedure with the patient in these positions should be as short as feasibly possible (59). Appropriate patient positioning will also help to prevent pressure ulcers, skin irritation, burns, nerve damage, circulatory problems and hypothermia. Positioning injuries can affect the skin, soft tissues, joints, ligaments and bones as well as the eyes, nerves and blood vessels. The risk of pressure sores can be minimised by utilising pressure-relieving aids on operating tables (60).

All members of the surgical team must maintain vigilance regarding patient positioning and other factors that may contribute to perioperative risk. The nursing team plays a pivotal role in continuously monitoring the position and movement of laparoscopic arms and trocars throughout the procedure and should be encouraged to speak up immediately if any unintended contact or pressure is identified (61). For surgeries anticipated to last longer, implementing a second surgical timeout to reassess patient positioning has been proposed as an effective strategy to mitigate positioning-related complications (62).

### 5.3 Optimising temperature

Intraoperative hypothermia (<36.0 °C) is a common consequence of anaesthesia, which increases morbidity and potentially increases mortality.^3^ Athletes often exhibit distinct physiological characteristics, including a higher surface-area-to-mass ratio and lower body fat percentage, both of which can increase heat loss in cold environments (63). As a result, certain athletes may be more susceptible to hypothermia, particularly during prolonged exposure or post-surgical recovery in cool environments. Heat loss occurs in three phases. The first phase (caused by the redistribution of heat) is responsible for the greatest fall in body temperature (64). Operations occurring under general anaesthesia have a higher risk for causing hypothermia compared to awake surgery e.g., under spinal anaesthesia. The risk of intraoperative and post operative hypothermia can be minimised by regular temperature measurements, use of warmed intravenous fluids and use of forced-air warming devices (such as a Bair Hugger) (65). Additional measures may also be employed, including the use of warmed irrigation fluids, circulating water mattresses, and carbon-fibre resistive heating systems (66).

### 5.4 Avoidance of anaesthetic and early post-operative complications

Minimising early post-operative complications is essential, as even minor delays can significantly disrupt an athlete's training schedule and competition readiness. Therefore, the anaesthetist's perioperative management must aim to limit the likelihood of such complications. Elite athletes undergoing gynaecological surgery are a high-risk group for postoperative nausea and vomiting. Both gynaecological and laparoscopic surgeries are significant risk factors (67). For this reason, the anaesthetist should mitigate potential risks (e.g., minimise the use of volatile anaesthesia, consider regional or total intravenous anaesthesia and, where possible, avoid postoperative opioids) and if the patient has at least two risk factors for postoperative nausea and vomiting, 3–4 antiemetic agents of different drug classes should be given as prophylaxis (68).

Central neuraxial blockade may facilitate surgery whilst avoiding the need for, and complications associated with, general anaesthesia. Additionally, peripheral nerve blockade may contribute to a multimodal, opiate-sparing analgesic regime irrespective of anaesthetic choice. Although these nerve blocks may provide optimal anaesthesia and/or analgesia, their benefits must be balanced with the risk of neuropathy. Although uncommon (central neuraxial blockade: 0.002–0.004% risk of permanent injury (69), peripheral nerve block: 1% risk of sensorimotor disturbance at 2 weeks or 0.03% risk of symptoms at 1 year (70), even temporary sensory or motor neuropathy may significantly impact one's RTP and represent a meaningful risk to an elite athlete. Therefore, these data, the location of the nerve block and the intended benefits should be considered and discussed with the patient during the informed consent process. Suxamethonium-induced postoperative myalgia, which can be severe and limit recovery, is more common in younger patients with greater muscle mass and should therefore be avoided (71).

Postoperative pulmonary complications (PPCs) are common, especially after major surgery, and are associated with significant morbidity and mortality. Although there is no universal definition for lung protective ventilation strategies, which can reduce the incidence of PPCs, breathing mechanics and respiratory function will be improved by individualising mechanical ventilation parameters. Suggested initial ventilator settings include a tidal volume of 6–8 ml/kg of predicted body weight and 5 cm H20 of positive end-expiratory pressure which should subsequently be individualized (72).

## 6 Post-operative management

Post-operative management plays a central role in facilitating safe and effective recovery following gynaecological surgery in elite female athletes. This period must be approached with a structured, individualised, and multidisciplinary plan that accounts for the short-, medium-, and long-term phases of rehabilitation (Figure 1).

### 6.1 Post-operative recovery and rehabilitation

In the short term, the clinician should ensure the athlete has an appropriate post-operative debrief and surgical review on Day One after the procedure. This allows the athlete's questions about surgery to be answered and for the identification of any early post-operative complications. If a longer than 24 h stay in hospital is required, the use of incentive spirometry can be used as an adjunct to reduce the risk of PPCs (73).

The medium to long term rehabilitation of the athlete should be tailored to their individual needs and goals, with a focus on optimising pain relief, restoring pelvic floor function, core stability, and overall musculoskeletal strength and flexibility. Enhanced recovery after surgery (ERAS) programmes have become the gold standard of care in many surgical specialties (74). A recent meta-analysis demonstrated that ERAS pathways significantly reduce length of hospital stay without increasing readmission rates or rates of ileus across benign and oncological gynaecological surgery (75). ERAS has a specific post-operative focus on early nourishment, planned mobilisation, early removal of catheters and wound drains, and regular analgesia (76).

### 6.2 Pain management

Postoperative pain management should be done in consultation with the anaesthetist. Ideally opiates should be minimised to avoid the risk of nausea, vomiting or ileus, and the clinicians should be aware of WADA regulations with regards to The Prohibited List and the potential need for TUEs. The IOC's consensus statement on pain management in elite athletes specifically highlights non-pharmacological pain management including pain education, psychosocial interventions and physiotherapy. This may include adjuncts such as transcutaneous electrical nerve stimulation (TENS) (77).

### 6.3 The multidisciplinary team

The medium to long term approach to recovery will require specialists in physiotherapy, nutrition, sleep, and sports psychology. Physiotherapy input is essential to provide a personalised recovery training plan to enable safe return to training. Historically, there has been significant variation in the availability of physiotherapy for patients after gynaecological surgery and in the regimens practised by physiotherapists. However, the recent robust evidence supporting ERAS has highlighted the importance of targeted physiotherapist input after major surgery (78).

More focus has been given to nutrition and sleep in recovery and performance in recent years, with evidence that sleep deprivation has a detrimental impact on healing (79). Similarly, nutrition must be maintained to minimise muscle loss and inadequate dietary protein and energy intake has been associated with loss of muscle mass in the postoperative period (80). A sports psychologist should be considered for those athletes who need further input due to the mental aspects associated with a prolonged time out from training and competing (81).

## 7 How to optimise specific surgical modalities

The preceding sections have explored how each phase of preoperative, intraoperative, and postoperative management can be adapted to meet the specific needs of female athletes. The following section outlines key considerations for each of the main gynaecological surgical modalities—hysteroscopic, laparoscopic, and open surgery.

### 7.1 Hysteroscopic surgery

Beyond the general considerations already discussed in the paper, the gynaecologist performing a hysteroscopy on an elite athlete is unlikely to require specific modifications. As with all hysteroscopies, the aim to complete it in the outpatient (office) setting will improve recovery time, avoiding the need for a general anaesthetic, but the decision should ultimately be made based on patient choice after relevant counselling.^4^ Careful consideration should also be given to the choice of approach. Vaginoscopic technique is preferable for hysteroscopies, as it is faster, associated with less pain, and reduces the risk of inducing a vasovagal reaction compared to the traditional method, which involves the use of a vaginal speculum and cervical instrumentation (82). Hysteroscopy resection of lesions under direct vision, rather than blind polypectomy, is preferred to reduce the risk of recurrence and need for repeat procedures (83). Hysteroscopic procedures requiring a general anaesthetic should be managed with appropriate diligence paid to the pre-, intra- and post-operative factors already described.

### 7.2 Laparoscopic surgery

Laparoscopy remains the standard of care for most benign and many oncological gynaecological procedures, offering reduced tissue trauma, lower infection rates, and quicker return to activity (84). However, unique anatomical and physiological considerations in athletes must inform surgical technique and access. On average, athletes have lower body mass indexes compared to the general population (85). In thin patients, the distance from the anterior abdominal wall to the retroperitoneal vascular structures can be as little as 2 cm (86). The Royal College of Obstetricians and Gynaecologists’ Green-top Guideline recommends open entry or use of Palmer's point in women with a low BMI to reduce the risk of posterior abdominal wall vascular injury (87).^5^

There are methods to reduce post-laparoscopy shoulder-tip pain (STP). A specific technique for releasing the pneumoperitoneum (pulmonary recruitment manoeuvre, extended assisted ventilation or actively aspirating intra-abdominal gas) has been shown to reduce STP. Intraperitoneal fluid instillation, the use of an intraperitoneal drain and local anaesthetic applied to the peritoneal cavity (not subdiaphragmatic) are also methods supported by data from RCTs (88).

Port number and placement should be carefully planned, with the use of the minimum number of trocars needed to maintain surgical safety and ergonomics. There does not seem to be an advantage in single-port laparoscopy vs. conventional laparoscopy regarding pain nor hospital stay (89). For athletes, conventional multi-port access with meticulous fascial closure should remain the standard. Attention should be given to the restoration of abdominal wall anatomy in the port sites to minimise the risk of postoperative hernias and muscle weakness. Particular attention should be paid to closure of the abdominal wall at port sites ≥10 mm, to reduce the risk of port-site herniation (90). Notably, an imbalance in muscle strength, balance, stability, or endurance, particularly between a weaker abdominal wall and a stronger hip adductor muscle group, may increase stress on the inguinal region and contribute to the development of a sports hernia (91). The risk of hernias is greater with 10 mm ports, so laparoscopy with only 5 mm ports, including for the camera, should be considered (92). The surgeon should consider standardized advice on restricting high-load exercises before fascial healing is radiologically or clinically confirmed (93).

In athletes for whom abdominal aesthetics are a priority, such as those in sports with exposed midriff attire, consideration may be given to fine suture techniques or Steri-strip closure only of small laparoscopic port sites to minimise visible scarring (94).

### 7.3 Open surgery

While laparoscopic approaches are preferred, there, of course, remain clinical situations where open surgery is indicated or unavoidable. If laparoscopic approaches to surgery are not acceptable and open surgery needs to be performed, techniques to reduce healing time and complications from wound healing should be considered. A mini laparotomy can be performed where appropriate. This involves a smaller incision measuring 2 to 4 cm through which an ovarian cyst, for example, can be aspirated and removed (95, 96). A smaller incision can reduce healing time but should not be used at the expense of increasing the difficulty with access during an open surgery. Incisional hernias after laparotomy are associated with significant morbidity. Recent research on prevention of incisional hernia formation suggests that a laparotomy closure technique using a slowly absorbable monofilament suture may be effective in lowering morbidity (86).

## 8 Discussion

The surgical management of elite female athletes presents a unique challenge that extends beyond the traditional goals of peri-operative care. While the primary objective of surgery remains the safe and effective treatment of gynaecological pathology, clinicians must simultaneously consider the athlete's performance goals, training schedules, contractual obligations, psychological wellbeing, and long-term sporting career. This review highlights the need for an individualised and multidisciplinary approach that integrates principles from gynaecology, sports medicine, anaesthesia, physiotherapy, nutrition, and psychology to optimise both clinical and performance outcomes.

A recurring theme throughout this review is the substantial gap between the increasing participation of women in elite sport and the limited evidence available to guide their surgical care. Although many of the peri-operative principles discussed are supported by evidence from general surgical populations, relatively few studies have specifically evaluated elite female athletes undergoing gynaecological procedures. Consequently, much of the current guidance relies on extrapolation from broader surgical, rehabilitation, and sports medicine literature. While such extrapolation is necessary given the scarcity of athlete-specific evidence, it should not be viewed as a substitute for direct research and high quality evidence from robust prospective studies. Elite athletes differ from the general population in several important respects and these factors may influence peri-operative risk, recovery trajectories, and RTP outcomes in ways that are not adequately captured by studies conducted in non-athletic populations.

The review also highlights several unresolved clinical challenges. One of the most important is the absence of procedure-specific RTP data following gynaecological surgery. Current recommendations regarding recovery timelines after hysteroscopic, laparoscopic, and open procedures are largely based on expert opinion or extrapolation from general rehabilitation principles. However, RTP decisions in elite sport carry significant consequences, and both premature return and excessive restriction may negatively affect athletic performance and career progression. Prospective studies evaluating functional recovery and sport-specific outcomes following common gynaecological procedures are therefore urgently needed.

Another important gap relates to ERAS pathways. ERAS programmes have transformed peri-operative care across multiple surgical specialties and are associated with reduced complications, shorter hospital stays, and faster recovery. However, existing ERAS protocols have been developed for general patient populations and do not specifically address the physiological and performance demands of elite athletes. Future research should evaluate whether athlete-adapted ERAS pathways, incorporating sport-specific nutritional strategies, accelerated rehabilitation programmes, and performance-focused recovery metrics, can further improve outcomes in this population.

Pain management represents another area requiring further investigation. Contemporary peri-operative practice increasingly favours opioid-sparing analgesic strategies because of concerns regarding side effects, delayed mobilisation, and impaired recovery. In elite athletes, these concerns are compounded by anti-doping regulations and restrictions imposed by the World Anti-Doping Agency (WADA). However, the desire to minimise opioid exposure must be balanced against the need to provide adequate postoperative analgesia. Insufficient pain control may impair rehabilitation, disrupt sleep, delay recovery, and negatively affect psychological wellbeing. The optimal balance between effective analgesia and compliance with anti-doping regulations remains uncertain and warrants further study.

Several limitations of this review should be acknowledged. As a narrative review, it does not employ the formal study selection, quality assessment, and evidence synthesis methods used in systematic reviews. However, the current literature base is not sufficiently mature to support a comprehensive systematic review, as athlete-specific studies are scarce and the available evidence is heterogeneous. Consequently, strict inclusion and exclusion criteria would likely have excluded much of the clinically relevant literature and limited the practical applicability of the review. To address this challenge, evidence from related fields, including surgery, anaesthesia, rehabilitation, and sports medicine, was incorporated where appropriate and carefully interpreted within the context of elite female athletes. Nevertheless, the strength of evidence supporting individual recommendations varies considerably and should be interpreted accordingly. We also acknowledge the homogeneous use of the term “elite female athlete”, as athletes in high-impact, aesthetic, endurance, strength-based and team sports will have distinct baseline risk profiles, intra-abdominal pressure demands, pelvic floor vulnerabilities, recovery trajectories and RTP requirements. Peri-operative planning should therefore be individualised according to the athlete's sporting discipline, loading pattern, competitive calendar and performance demands.

Ultimately, the increasing visibility and participation of women in elite sport demands a corresponding evolution in clinical research and peri-operative care. The framework presented in this review provides a pragmatic approach to current practice while highlighting the significant evidence gaps that remain. Future prospective studies, athlete registries, and multidisciplinary collaborations will be essential to generate high-quality evidence capable of informing athlete-specific peri-operative guidelines, rehabilitation pathways, and RTP recommendations.

## Footnotes

^1^Inside FIFA. *Setting the Pace: FIFA Benchmarking Report* 4th Edition. https://inside.fifa.com/womens-football/fifa-benchmarking-report [Accessed June 5, 2026].

^2^National Institute for Health and Care Excellence. *Perioperative care in adults* (2020). https://www.nice.org.uk/guidance/ng180 [Accessed January 16, 2025].

^3^National Institute for Health and Care Excellence. *Hypothermia: prevention and management in adults having surgery*(2008). https://www.nice.org.uk/guidance/cg65 [Accessed January 16, 2025].

^4^Royal College of Obstetricians and Gynaecologists. *Outpatient Hysteroscopy: Green-top Guideline No. 59*. https://www.rcog.org.uk/guidance/browse-all-guidance/green-top-guidelines/outpatient-hysteroscopy-green-top-guideline-no-59/ [Accessed January 16, 2025].

^5^Royal College of Obstetricians and Gynaecologists. *Laparoscopic Injuries: Green-top Guideline No. 49*. https://www.rcog.org.uk/guidance/browse-all-guidance/green-top-guidelines/laparoscopic-injuries-green-top-guideline-no-49/ [Accessed January 16, 2025].

## References

[1] Lebel K Mumcu C Pegoraro A LaVoi NM Lough N Antunovic D. Re-thinking women’s sport research: looking in the mirror and reflecting forward. Front Sports Act Living. (2021) 3:746441. 10.3389/fspor.2021.746441 34708200 PMC8542874

[2] L’Heveder A Chan M Mitra A Kasaven L Saso S Prior T. Sports obstetrics: implications of pregnancy in elite sportswomen, a narrative review. J Clin Med. (2022) 11:4977. 10.3390/jcm11174977 36078907 PMC9456821

[3] Ramagole D van Rensburg DCJ , Cowie C Mehta R Ramkilawon G Pluim BM. Gynaecological health patterns and motherhood experiences of female professional football players. 10.1101/2024.08.26.24312465 PMC11855395 40003362

[4] Dave SC Fisher M. Relative energy deficiency in sport (RED - S). Curr Probl Pediatr Adolesc Health Care. (2022) 52:101242. 10.1016/j.cppeds.2022.101242 35915044

[5] Jones BP L’Heveder A Saso S Yazbek J Smith JR Dooley M. Sports gynaecology. Obstet Gynaecol. (2019) 21:85–94. 10.1111/tog.12557

[6] Culleton-Quinn E Bø K Fleming N Mockler D Cusack C Daly D. Elite female athletes’ experiences of symptoms of pelvic floor dysfunction: a systematic review. Int Urogynecology J. (2022) 33:2681–711. 10.1007/s00192-022-05302-6 PMC9477953 36040507

[7] Teixeira RV Colla C Sbruzzi G Mallmann A Paiva LL. Prevalence of urinary incontinence in female athletes: a systematic review with meta-analysis. Int Urogynecology J. (2018) 29:1717–25. 10.1007/s00192-018-3651-1 29654349

[8] Louis-Charles K Biggie K Wolfinbarger A Wilcox B Kienstra CM. Pelvic floor dysfunction in the female athlete. Curr Sports Med Rep. (2019) 18:49–52. 10.1249/JSR.0000000000000563 30730341

[9] Romero-Franco N Fernández-Domínguez JC Vico-Moreno E Bachero-Mena B Sastre-Munar A Bosch-Donate E. Effects of a 12-weeks pelvic floor exercises program on sports performance of female athletes: an exploratory randomized clinical trial. Phys Ther Sport. (2026) 78:101898. 10.1016/j.ptsp.2026.101898 41671735

[10] Patel DR Omar H Terry M. Sport-related performance anxiety in young female athletes. J Pediatr Adolesc Gynecol. (2010) 23:325–35. 10.1016/j.jpag.2010.04.004 20869282

[11] Mann BJ Grana WA Indelicato PA O’Neill DF George SZ. A survey of sports medicine physicians regarding psychological issues in patient-athletes. Am J Sports Med. (2007) 35:2140–7. 10.1177/0363546507304140 17641103

[12] Dergaa I Ben Saad H El Omri A Duque JDP Chaabane M Chamari K. Mental, physiological and medical considerations for elite football players in the Saudi pro league: a call for action. BMJ Open Sport Exerc Med. (2023) 9:e001789. 10.1136/bmjsem-2023-001789 37953968 PMC10632892

[13] Brewer BW Chatterton HA. Athletic identity and sport injury processes and outcomes in young athletes: a supplemental narrative review. J Funct Morphol Kinesiol. (2024) 9:191. 10.3390/jfmk9040191 39449485 PMC11503344

[14] Thiemann P Legenbauer T Vocks S Platen P Auyeung B Herpertz S. Eating disorders and their putative risk factors among female German professional athletes. Eur Eat Disord Rev. (2015) 23:269–76. 10.1002/erv.2360 25828261

[15] Harrison SE Wilson F Ackerman KE Hoegh M Brown N. Period pain in female athletes: a call to action - time to fill the evidence gap and develop tailored management pathways. Br J Sports Med. (2026) bjsports-2025-110565. 10.1136/bjsports-2025-110565 42167895

[16] Singh SS Gude K Perdeaux E Gattrell WT Becker CM. Surgical outcomes in patients with endometriosis: a systematic review. J Obstet Gynaecol Can. (2020) 42:881–888.e11. 10.1016/j.jogc.2019.08.004 31718952

[17] Dijkstra HP Pollock N Chakraverty R Ardern CL. Return to play in elite sport: a shared decision-making process. Br J Sports Med. (2017) 51:419–20. 10.1136/bjsports-2016-096209 27474390

[18] Nelis S Dijkstra HP Damman OC Farooq A Verhagen E. Shared decision-making with athletes: a survey study of healthcare professionals’ perspectives. BMJ Open Sport Exerc Med. (2024) 10:e001913. 10.1136/bmjsem-2024-001913 38736642 PMC11086382

[19] Recker F Gembruch U Strizek B. Clinical ultrasound applications in obstetrics and gynecology in the year 2024. J Clin Med. (2024) 13:1244. 10.3390/jcm13051244 38592066 PMC10931841

[20] Landolfo C Bourne T Froyman W Van Calster B Ceusters J Testa AC. Benign descriptors and ADNEX in two-step strategy to estimate risk of malignancy in ovarian tumors: retrospective validation in IOTA5 multicenter cohort. Ultrasound Obstet Gynecol. (2023) 61:231–42. 10.1002/uog.26080 36178788 PMC10107772

[21] Heremans R Wynants L Valentin L Leone FPG Pascual MA Fruscio R. Estimating risk of endometrial malignancy and other intracavitary uterine pathology in women without abnormal uterine bleeding using IETA-1 multinomial regression model: validation study. Ultrasound Obstet Gynecol. (2024) 63:556–63. 10.1002/uog.27530 37927006

[22] Van den Bosch T Dueholm M Leone FPG Valentin L Rasmussen CK Votino A. Terms, definitions and measurements to describe sonographic features of myometrium and uterine masses: a consensus opinion from the morphological uterus sonographic assessment (MUSA) group. Ultrasound Obstet Gynecol. (2015) 46:284–98. 10.1002/uog.14806 25652685

[23] Indrielle-Kelly T Frühauf F Fanta M Burgetova A Lavu D Dundr P. Application of international deep endometriosis analysis (IDEA) group consensus in preoperative ultrasound and magnetic resonance imaging of deep pelvic endometriosis. Ultrasound Obstet Gynecol. (2020) 56:115–6. 10.1002/uog.21960 31876340

[24] McNulty KL Elliott-Sale KJ Dolan E Swinton PA Ansdell P Goodall S. The effects of menstrual cycle phase on exercise performance in eumenorrheic women: a systematic review and meta-analysis. Sports Med. (2020) 50:1813–27. 10.1007/s40279-020-01319-3 32661839 PMC7497427

[25] Martínez-Fortuny N Alonso-Calvete A Da Cuña-Carrera I Abalo-Núñez R. Menstrual cycle and sport injuries: a systematic review. Int J Environ Res Public Health. (2023) 20:3264. 10.3390/ijerph20043264 36833966 PMC9958828

[26] Carli F Scheede-Bergdahl C. Prehabilitation to enhance perioperative care. Anesthesiol Clin. (2015) 33:17–33. 10.1016/j.anclin.2014.11.002 25701926

[27] Khuri SF Henderson WG DePalma RG Mosca C Healey NA Kumbhani DJ. Participants in the VA national surgical quality improvement program. Determinants of long-term survival after major surgery and the adverse effect of postoperative complications. Ann Surg. (2005) 242:326–41; discussion 341-343. 10.1097/01.sla.0000179621.33268.83 16135919 PMC1357741

[28] Wynter-Blyth V Moorthy K. Prehabilitation: preparing patients for surgery. Br Med J. (2017) 358:j3702. 10.1136/bmj.j3702 28790033

[29] National Institute for Health and Care Excellence. Routine preoperative tests for elective surgery. NICE guideline NG45. London: NICE; 2016. Available at: https://www.nice.org.uk/guidance/ng45. (Accessed January 16, 2025). 41643037

[30] Fitzpatrick D Leckie T Heine G Hodgson L. The use of pain killers (NSAIDs) in athletes: how large is the risk? J Sci Med Sport. (2025) 28:198–205. 10.1016/j.jsams.2024.11.010 39665963

[31] Sim M Garvican-Lewis LA Cox GR Govus A McKay AKA Stellingwerff T. Iron considerations for the athlete: a narrative review. Eur J Appl Physiol. (2019) 119:1463–78. 10.1007/s00421-019-04157-y 31055680

[32] Peeling P Blee T Goodman C Dawson B Claydon G Beilby J. Effect of iron injections on aerobic-exercise performance of iron-depleted female athletes. Int J Sport Nutr Exerc Metab. (2007) 17:221–31. 10.1123/ijsnem.17.3.221 17693684

[33] Castell LM Nieman DC Bermon S Peeling P. Exercise-induced illness and inflammation: can immunonutrition and iron help? Int J Sport Nutr Exerc Metab. (2019) 29:181–8. 10.1123/ijsnem.2018-0288 30507260

[34] Peake J Nosaka K Suzuki K. Characterization of inflammatory responses to eccentric exercise in humans. Exerc Immunol Rev. (2005) 11:64–85. PMID: 16385845. 16385845

[35] Volek JS Forsythe CE Kraemer WJ. Nutritional aspects of women strength athletes. Br J Sports Med. (2006) 40:742–8. 10.1136/bjsm.2004.016709 16855068 PMC2564387

[36] Gillis C Wischmeyer PE. Pre-operative nutrition and the elective surgical patient: why, how and what? Anaesthesia. (2019) 74:27–35. 10.1111/anae.14506 30604414

[37] Bonci CM Bonci LJ Granger LR Johnson CL Malina RM Milne LW. National athletic Trainers’ association position statement: preventing, detecting, and managing disordered eating in athletes. J Athl Train. (2008) 43:80–108. 10.4085/1062-6050-43.1.80 18335017 PMC2231403

[38] Campbell B Kreider RB Ziegenfuss T La Bounty P Roberts M Burke D. International society of sports nutrition position stand: protein and exercise. J Int Soc Sports Nutr. (2007) 4:8. 10.1186/1550-2783-4-8 17908291 PMC2117006

[39] Kratzing C. Pre-operative nutrition and carbohydrate loading. Proc Nutr Soc. (2011) 70:311–5. 10.1017/S0029665111000450 21781358

[40] Zivkovic AM Telis N German JB Hammock BD. Dietary omega-3 fatty acids aid in the modulation of inflammation and metabolic health. Calif Agric. (2011) 65:106–11. 10.3733/ca.v065n03p106 PMC4030645 24860193

[41] Voulgaridou G Papadopoulou SK Detopoulou P Tsoumana D Giaginis C Kondyli FS. Vitamin D and calcium in osteoporosis, and the role of bone turnover markers: a narrative review of recent data from RCTs. Diseases. (2023) 11:29. 10.3390/diseases11010029 36810543 PMC9944083

[42] Pollock N Dijkstra P Chakraverty R Hamilton B. Low 25(OH) vitamin D concentrations in international UK track and field athletes. South Afr J Sports Med. (2012) 24:55–9. 10.17159/2078-516X/2012/v24i2a336

[43] Pollock N Chakraverty R Taylor I Killer SC. An 8-year analysis of magnesium Status in elite international track & field athletes. J Am Coll Nutr. (2020) 39:443–9. 10.1080/07315724.2019.1691953 31829845

[44] Gombart AF Pierre A Maggini S. A review of micronutrients and the immune system–working in harmony to reduce the risk of infection. Nutrients. (2020) 12:236. 10.3390/nu12010236 31963293 PMC7019735

[45] Lygizos V Haidopoulos D Vlachos DE Varthaliti A Fanaki M Daskalakis G. Immunonutrition in ERAS protocol for patients with gynecologic cancer: a narrative review of the literature. Life. (2025) 15:487. 10.3390/life15030487 40141831 PMC11943961

[46] Ma X Pei B Wu N Wang C Yu Y Yang W. Current research and future prospects of immunonutrition in gastrointestinal malignancies. Front Immunol. (2024) 15:1420415. 10.3389/fimmu.2024.1420415 39308867 PMC11412812

[47] Nazha B Pandya B Spyropoulos AC Kessler CM. Treatment of venous thromboembolism in elite athletes: a suggested approach to individualized anticoagulation. Semin Thromb Hemost. (2018) 44:813–22. 10.1055/s-0038-1673690 30296792

[48] Swan D Carter-Brzezinski L Thachil J. Management of venous thromboembolism in athletes. Blood Rev. (2021) 47:100780. 10.1016/j.blre.2020.100780 33229140

[49] Zhou NJ Kamil RJ Hillel AT Tan M Walsh J Russell JO. The role of preoperative briefing and postoperative debriefing in surgical education. J Surg Educ. (2021) 78:1182–8. 10.1016/j.jsurg.2020.11.001 33257299

[50] Bourgonjon B Vermeylen K Tytgat N Forget P. Anaesthesia for elite athletes. Eur J Anaesthesiol EJA. (2022) 39:825. 10.1097/EJA.0000000000001719 35943185

[51] The World Anti-Doping Code. World Anti Doping Agency. Available online at: https://www.wada-ama.org/en/what-we-do/world-anti-doping-code (Accessed January 16, 2025).

[52] Therapeutic Use Exemptions (TUEs). World Anti Doping Agency. Available online at: https://www.wada-ama.org/en/athletes-support-personnel/therapeutic-use-exemptions-tues (Accessed January 16, 2025).

[53] The Prohibited List. World Anti Doping Agency. Available online at: https://www.wada-ama.org/en/prohibited-list (Accessed April 14, 2025).

[54] Warner MA Warner DO Harper CM Schroeder DR Maxson PM. Lower extremity neuropathies associated with lithotomy positions. Anesthesiology. (2000) 93:938–42. 10.1097/00000542-200010000-00010 11020742

[55] Bjøro B Mykkeltveit I Rustøen T Candas Altinbas B Røise O Bentsen SB. Intraoperative peripheral nerve injury related to lithotomy positioning with steep trendelenburg in patients undergoing robotic-assisted laparoscopic surgery - A systematic review. J Adv Nurs. (2020) 76:490–503. 10.1111/jan.14271 31736124

[56] Fleisch MC Bader W Balzer K Bennefeld L Boeing C Bremerich D. The prevention of positioning injuries during gynecologic surgery. Guideline of the DGGG, OEGGG and SGGG (S2k level, AWMF registry number 015/077, October 2020). Geburtshilfe Frauenheilkd. (2021) 81:447–68. 10.1055/a-1378-4209 33867563 PMC8046520

[57] Hay AK McDougall A Hinstridge P Rajakuldendran S Yoong W. Prolonged brachial plexus neuropathy: a rare complication following protracted endometriosis surgery in lloyd-davies position. BMJ Case Rep. (2021) 14:e243408. 10.1136/bcr-2021-243408 34844958 PMC8634370

[58] Ripa M Schipa C Kopsacheilis N Nomikarios M Perrotta G De Rosa C. The impact of steep trendelenburg position on intraocular pressure. J Clin Med. (2022) 11:2844. 10.3390/jcm11102844 35628970 PMC9146028

[59] Romanowski L Reich H McGlynn F Adelson MD Taylor PJ. Brachial plexus neuropathies after advanced laparoscopic surgery. Fertil Steril. (1993) 60:729–32. 10.1016/s0015-0282(16)56233-5 8405536

[60] Primiano M Friend M McClure C Nardi S Fix L Schafer M. Pressure ulcer prevalence and risk factors among prolonged surgical procedures in the OR. AORN J. (2011) 94:555–66. 10.1016/j.aorn.2011.03.014 22118201 PMC4467017

[61] Wevling A Linqvist Leonardsen AC. Positioning the surgical patient – roles, responsibilities and challenges. A qualitative study. J Adv Nurs. (2025) 81:968–77. 10.1111/jan.16278 38888365 PMC11730183

[62] Song JB Vemana G Mobley JM Bhayani SB. The second “time-out”: a surgical safety checklist for lengthy robotic surgeries. Patient Saf Surg. (2013) 7:19. 10.1186/1754-9493-7-19 23731776 PMC3689613

[63] Falk B Dotan R. Temperature regulation and elite young athletes. Med Sport Sci. (2011) 56:126–49. 10.1159/000320645 21178371

[64] Díaz M Becker DE. Thermoregulation: physiological and clinical considerations during sedation and general anesthesia. Anesth Prog. (2010) 57:25–33. 10.2344/0003-3006-57.1.25 20331336 PMC2844235

[65] Xu H Wang Z Lu Y Guan X Ma Y Malone DC. Value of active warming devices for intraoperative hypothermia prevention—a meta-analysis and cost-benefit analysis. Int J Environ Res Public Health. (2021) 18:11360. 10.3390/ijerph182111360 34769877 PMC8582721

[66] Simegn GD Bayable SD Fetene MB. Prevention and management of perioperative hypothermia in adult elective surgical patients: a systematic review. Ann Med Surg. (2021) 72:103059. 10.1016/j.amsu.2021.103059 PMC8605381 34840773

[67] Apfel CC Heidrich FM Jukar-Rao S Jalota L Hornuss C Whelan RP. Evidence-based analysis of risk factors for postoperative nausea and vomiting. Br J Anaesth. (2012) 109:742–53. 10.1093/bja/aes276 23035051

[68] Gan TJ Belani KG Bergese S Chung F Diemunsch P Habib AS. Fourth consensus guidelines for the management of postoperative nausea and vomiting. Anesth Analg. (2020) 131:411–48. 10.1213/ANE.0000000000004833 32467512

[69] Cook TM Counsell D Wildsmith JAW. Royal college of anaesthetists third national audit project. Major complications of central neuraxial block: report on the third national audit project of the royal college of anaesthetists. Br J Anaesth. (2009) 102:179–90. 10.1093/bja/aen360 19139027

[70] Lemke E Johnston DF Behrens MB Seering MS McConnell BM Swaran Singh TS. Neurological injury following peripheral nerve blocks: a narrative review of estimates of risks and the influence of ultrasound guidance. Reg Anesth Pain Med. (2024) 49:122–32. 10.1136/rapm-2023-104855 37940348

[71] Chekol B Zurbachew N Mekete G Bayuh E Teshome D. Prevalence and associated factors of postoperative suxamethonium-induced myalgia in surgical patients at debre tabor comprehensive specialized hospital Ethiopia: a cross-sectional study. Sci Rep. (2024) 14:16552. 10.1038/s41598-024-65779-7 39019942 PMC11255331

[72] Young CC Harris EM Vacchiano C Bodnar S Bukowy B Elliott RRD. Lung-protective ventilation for the surgical patient: international expert panel-based consensus recommendations. Br J Anaesth. (2019) 123:898–913. 10.1016/j.bja.2019.08.017 31587835

[73] Eltorai AEM Szabo AL Antoci V Ventetuolo CE Elias JA Daniels AH. Clinical effectiveness of incentive spirometry for the prevention of postoperative pulmonary complications. Respir Care. (2018) 63:347–52. 10.4187/respcare.05679 29279365

[74] Zaed I Bossi B Ganau M Tinterri B Giordano M Chibbaro S. Current state of benefits of enhanced recovery after surgery (ERAS) in spinal surgeries: a systematic review of the literature. Neurochirurgie. (2022) 68:61–8. 10.1016/j.neuchi.2021.04.007 33901525

[75] O’Neill AM Calpin GG Norris L Beirne JP. The impact of enhanced recovery after gynaecological surgery: a systematic review and meta-analysis. Gynecol Oncol. (2023) 168:8–16. 10.1016/j.ygyno.2022.10.019 36356373

[76] Torbé E Crawford R Nordin A Acheson N. Enhanced recovery in gynaecology. Obstet Gynaecol. (2013) 15:263–8. 10.1111/tog.12061

[77] Rugg CM Ginder JH Bharadwaj A Vomer R Dale GA Ketterly J. Perioperative management in the collegiate athlete: an integrated approach. Sports Med Int Open. (2023) 7:E1–8. 10.1055/a-2051-7756 37101550 PMC10125641

[78] Boden I. Physiotherapy management of major abdominal surgery. J Physiother. (2024) 70:170–80. 10.1016/j.jphys.2024.06.005 38902197

[79] Sipilä RM Kalso EA. Sleep well and recover faster with less pain—a narrative review on sleep in the perioperative period. J Clin Med. (2021) 10:2000. 10.3390/jcm10092000 34066965 PMC8124518

[80] Hardy EJ Deane CS Lund JN Phillips BE. Loss of muscle mass in the immediate post-operative period is associated with inadequate dietary protein and energy intake. Eur J Clin Nutr. (2023) 77:503–5. 10.1038/s41430-023-01264-0 36702923 PMC10115623

[81] Herrero CP Jejurikar N Carter CW. The psychology of the female athlete: how mental health and wellness mediate sports performance, injury and recovery. Ann Jt. (2021) 6:38. 10.21037/aoj-20-53

[82] De Silva PM Carnegy A Smith PP Clark TJ. Vaginoscopy for office hysteroscopy: a systematic review & meta-analysis. Eur J Obstet Gynecol Reprod Biol. (2020) 252:278–85. 10.1016/j.ejogrb.2020.06.045 32645643

[83] Lee MMH. Endometrial polyp removed by a manual hysteroscopic tissue removal device. Gynecol Minim Invasive Ther. (2020) 9:34–5. 10.4103/GMIT.GMIT_116_18 32090011 PMC7008644

[84] Buia A Stockhausen F Hanisch E. Laparoscopic surgery: a qualified systematic review. World J Methodol. (2015) 5:238–54. 10.5662/wjm.v5.i4.238 26713285 PMC4686422

[85] Walsh J Heazlewood IT Climstein M. Body mass Index in master athletes: review of the literature. J Lifestyle Med. (2018) 8:79–98. 10.15280/jlm.2018.8.2.79 30474004 PMC6239137

[86] Bloemen A De Kleijn RJCMF Van Steensel S Aarts F Schreinemacher MHF Bouvy ND. Laparotomy closure techniques: do surgeons follow the latest guidelines? Results of a questionnaire. Int J Surg Lond Engl. (2019) 71:110–6. 10.1016/j.ijsu.2019.09.024 31561005

[87] Brierley G Arshad I Shakir F Visvathanan D Arambage K. Vascular injury during laparoscopic gynaecological surgery: a methodological approach for prevention and management. Obstet Gynaecol. (2020) 22:191–8. 10.1111/tog.12664

[88] Kaloo P Armstrong S Kaloo C Jordan V. Interventions to reduce shoulder pain following gynaecological laparoscopic procedures. Cochrane Database Syst Rev. (2019) 2019:CD011101. 10.1002/14651858.CD011101.pub2 PMC6353625 30699235

[89] Schmitt A Crochet P Knight S Tourette C Loundou A Agostini A. Single-Port laparoscopy vs conventional laparoscopy in benign adnexal diseases: a systematic review and meta-analysis. J Minim Invasive Gynecol. (2017) 24:1083–95. 10.1016/j.jmig.2017.07.001 28705751

[90] Deerenberg EB Henriksen NA Antoniou GA Antoniou SA Bramer WM Fischer JP. Updated guideline for closure of abdominal wall incisions from the European and American hernia societies. Br J Surg. (2022) 109:1239–50. 10.1093/bjs/znac302 36026550 PMC10364727

[91] Kopscik M Crisman JL Lomasney L Smith S Jadidi S. Sports hernias: a comprehensive review for clinicians. Cureus. (2023) 15:e43283. 10.7759/cureus.43283 37692688 PMC10492628

[92] Karthik S Augustine AJ Shibumon MM Pai MV. Analysis of laparoscopic port site complications: a descriptive study. J Minimal Access Surg. (2013) 9:59–64. 10.4103/0972-9941.110964 PMC3673575 23741110

[93] Güsgen C Willms A Schaaf S Prior M Weber C Schwab R. Lack of standardized advice on physical strain following abdominal surgery. Dtsch Ärztebl Int. (2020) 117:737–44. 10.3238/arztebl.2020.0737 33439823 PMC7878727

[94] Aitchison LP Chen AZL Toms C Sandroussi C Yeo DA Steffens D. To stitch or not to stitch: the skin closure of laparoscopic port sites, a meta-analysis. Surg Endosc. (2022) 36:7140–59. 10.1007/s00464-022-09269-9 35610480 PMC9485090

[95] Hicks-Courant K Awtrey CS Kim YB. Controlled decompression of large ovarian cystic tumors via mini-laparotomy using dermabond Advanced^TM^. Gynecol Oncol Rep. (2020) 32:100536. 10.1016/j.gore.2020.100536 32181316 PMC7063233

[96] Larson LE King NR. Minimally invasive approach to large ovarian cysts. J Minim Invasive Gynecol. (2024) 31:S54–5. 10.1016/j.jmig.2024.09.203

