# Supplementary material for: Optimising gynaecological surgical care for elite female athletes: a narrative review
Source: Front Sports Act Living. 2026 Jul 8;8:1746638. doi: 10.3389/fspor.2026.1746638 (PMC13388725; doi:10.3389/fspor.2026.1746638)
Supplement: Supplementary file 1 [file Table1.docx]

**Supplementary material**

**Table S1.** Databases, Search Dates, and Search Terms Used for the Literature Search

| **Database** | **Search Date** | **Search Terms** |
| --- | --- | --- |
| PubMed | August 2025 | ("elite athlete" OR "female athlete" OR athlete*) AND ("gynaecology" OR "gynecology" OR "gynaecological surgery" OR "gynecological surgery") AND ("peri-operative care" OR perioperative OR surgery OR rehabilitation OR "return to play" OR "sports medicine") |
| Embase | August 2025 | ('elite athlete' OR 'female athlete' OR athlete*) AND ('gynaecology' OR 'gynecology' OR 'gynaecological surgery' OR 'gynecological surgery') AND ('peri-operative care' OR perioperative OR surgery OR rehabilitation OR 'return to play' OR 'sports medicine') |
| Scopus | August 2025 | TITLE-ABS-KEY ("elite athlete" OR "female athlete" OR athlete*) AND ("gynaecology" OR "gynecology" OR "gynaecological surgery" OR "gynecological surgery") AND ("peri-operative care" OR perioperative OR surgery OR rehabilitation OR "return to play" OR "sports medicine") |

**Table S2.** Proposed Return-to-Play (RTP) Timelines Following Common Gynaecological Surgical Procedures in Elite Female Athletes.

| **Procedure** | **Extrapolated**  **RTP estimate** | **Evidence basis** |
| --- | --- | --- |
| Outpatient hysteroscopy | - Days to 1 week | RCOG [1] |
| Diagnostic laparoscopy | - 1–2 weeks for light training - 2–4 weeks for high-impact/contact sport | RCOG [2] |
| Laparoscopic cystectomy | - 2-4 weeks for light training - 4–6 weeks for high-impact/contact sport | RCOG [2]  Hong et al [3]  Mohammed et al [4] |
| Laparoscopic myomectomy | - 4–6 weeks* | Tsuzuki et al [5]  Peters et al [6]  Huff et al [7] |
| Laparoscopic hysterectomy | - 6-8 weeks for light training - 8+ weeks for high-impact/contact sport | Sanders et al [8]  Bouattour et al [9] |
| Open surgery / laparotomy | - 8-12+ weeks** | Rickett et al [10]  RCOG [11] |

* Depends on extend of myometrial involvement

** Individualised care based on indication and extend of laparotomy

+ Progression of physical activity should be gradual with more complex or major surgical procedures.

**References**

1. De Silva, P.M., et al., *Outpatient Hysteroscopy: (Green‐top Guideline no. 59)*. 2024, Wiley Online Library. p. e86-e110.

2. Well, R.R., *Information for You after a Laparoscopy.; 2015*.

3. Hong, Z., et al., *Effect of enhanced recovery after surgery on postoperativerecovery in ovarian cyst patients undergoing laparoscopic surgery.* Journal of Clinical Medicine in Practice, 2021. **25**(13): p. 60-63.

4. Mohammed, H.M., et al., *Quality of Recovery After Laparoscopic Ovarian Cystectomy: A Randomized Controlled Trial Comparing Opioid-Free Multimodal Analgesia Versus Opioid-Based Anesthesia.* Clin Ter, 2026. **177**(2): p. 249-257.

5. Tsuzuki, Y., et al., *Recovery of quality of life after laparoscopic myomectomy.* Journal of Obstetrics and Gynaecology Research, 2019. **45**(1): p. 176-181.

6. Peters, A., et al., *Enhanced recovery after surgery outcomes in minimally invasive nonhysterectomy gynecologic procedures.* American journal of obstetrics and gynecology, 2020. **223**(2): p. 234. e1-234. e8.

7. Huff, K.O., et al., *Returning to work after laparoscopic myomectomy: a prospective observational study.* Acta obstetricia et gynecologica Scandinavica, 2018. **97**(1): p. 68-73.

8. Sanders, A.P., et al., *Returning to work following minimally invasive hysterectomy.* Journal of Obstetrics and Gynaecology Canada, 2020. **42**(1): p. 80-83.

9. Bouattour, K., et al., *Recovery after low-impact laparoscopic hysterectomy: a randomized controlled clinical trial.* American Journal of Obstetrics and Gynecology, 2025.

10. Pickett, C.M., et al., *Surgical approach to hysterectomy for benign gynaecological disease.* Cochrane Database of Systematic Reviews, 2023(8).

11. Royal College of Obstetricians and Gynaecologists. Abdominal hysterectomy – recovering well [Internet]. London: RCOG; [cited 2026 Jun 7]. Available from: RCOG Abdominal Hysterectomy – Recovering Well
